# The mind's clock in sport: a narrative review of temporal processing across athletic disciplines

**DOI:** 10.3389/fspor.2026.1738879

**Published:** 2026-07-08

**Authors:** Olga Sysoeva, Victor Bolshakov, Oksana Galakhova, Evgeny Machnev, Maksim Markevich, Dina Mitiureva, Alena Ovakimian, Sergey Popov, Margarita Tсepelevich

**Affiliations:** 1 Centre for Research on Talent Development, Sirius University of Science and Technology, Sirius, Russia; 2 Institute of Higher Nervous Activity and Neurophysiology, Russian Academy of Sciences, Moscow, Russia; 3 OOO “Addon”, Moscow, Russia

**Keywords:** duration estimation, duration production, motor timing, sport, temporal processing, time perception, time-to-collision task

## Abstract

Time perception is a fundamental yet often underappreciated component of athletic performance. Athletes across various disciplines must continuously process temporal information to coordinate movements, anticipate events, and make effective decisions under pressure. This narrative review synthesizes current knowledge on temporal processing in sports without applying rigid methodological protocols, aiming to identify key patterns and sport-specific mechanisms. A literature search was conducted in PubMed and ScienceDirect for articles published between 2005 and 2025 using keywords related to time perception, sport types, and specific athletic disciplines. Thirty two studies met the inclusion criteria and were analyzed in detail. The reviewed paradigms included duration estimation, production, reproduction, discrimination, motor timing, representational momentum, temporal resolution and time-to-collision tasks. Two main findings emerge. First, distinct processing mechanisms for sub- and suprasecond intervals are consistently observed across paradigms and sport types: subsecond intervals tend to be overestimated/dilated, whereas suprasecond intervals are underestimated/shrunken. This pattern may reflect an adaptive strategy: temporal dilation of brief, critical events may preserve additional time for decision-making, although this mechanism appears limited to subsecond ranges. In contrast, compression of longer intervals may be more advantageous in extended play. Second, athletic experience modulates these effects. Engagement in sport is associated with enhanced suprasecond underestimation and subsecond dilation, particularly in high-stakes contexts where athletes frequently report subjective time slowing. These changes are sport-specific and often correlate with years of training. Physical exertion, fatigue, and emotional arousal systematically modulate temporal judgments, while accumulated expertise—rather than innate ability—appears to refine temporal processing through practice. These findings highlight time perception as a trainable, context-sensitive component of athletic expertise. Understanding how athletes perceive and utilize temporal information has implications for talent identification, training design, and performance optimization across sport disciplines.

## 1 Introduction

Time perception plays a critical role in athletic performance, influencing decision-making, reaction speed, and motor control under dynamically changing conditions. In the high-pressure environment of competitive sports, athletes are required to process temporal information rapidly and accurately – whether anticipating the trajectory of a ball, synchronizing movements with a teammate, or making split-second tactical decisions. Quantitative syntheses have confirmed that athletes outperform non-athletes in anticipation, automatic prediction, and decision-making (1–3). However, these meta-analyses mostly used isolated temporal processing paradigms or domain-general tests. Thus, the broader landscape of temporal processing across sport types and time scales remains relatively unexplored. Consequently, understanding how time is perceived and modulated in the sport context is essential for optimizing performance and training strategies. Throughout this review, we use the term ‘temporal processing’ to encompass both explicit time perception (e.g., duration estimation, reproduction, discrimination) and implicit timing abilities (e.g., motor synchronization, time-to-collision judgments, representational momentum).

When discussing temporal processing in sport-related settings, it is crucial to establish a clear conceptual framework to avoid misinterpretation—a common pitfall in time perception research (4). In this narrative review, we classify experimental evidence according to several key parameters.

The first parameter is the temporal scale as key events in sports unfold across diverse timescales. Some disciplines emphasize millisecond-level reactions and anticipation (e.g., returning a serve in tennis), while others require an ability to manage pacing strategy over seconds, minutes, and even hours (e.g., endurance running or rowing). In this respect, it is crucial to recognize that time perception mechanisms differ depending on the temporal scale, both at the cognitive and neural levels. A fundamental distinction exists between subsecond (<1 s) and suprasecond (>1 s) timing. Subsecond timing is thought to rely on automatic, sensory-motor processes that constitute discrete perceptual experiences, whereas suprasecond timing includes durations that are experienced as genuine temporal extensions; and an outer horizon near 10^2^ s marks the limit of direct duration experience, beyond which time is only cognitively reconstructed (5–8).

Second, temporal direction differentiates between judgments about past intervals and judgments about unfolding or future intervals (9, 10). Paradigms such as duration estimation and discrimination require participants to evaluate intervals that have already elapsed, while duration production and time-to-collision tasks involve anticipating or producing intervals in real time. This distinction is critical for interpretation: for example, subjective time acceleration leads to production of shorter durations but overestimation of elapsed time.

Third, we consider task specificity—whether the temporal judgment is made using conventional time units (e.g., seconds) or through implicit motor responses (e.g., tapping, anticipating a ball's arrival). This parameter allows us to integrate findings from diverse paradigms, including duration estimation, production, reproduction, discrimination, time-to-collision, motor timing, and temporal resolution tasks. We also consider the implicit measure of temporal prediction - representational momentum. It refers to the tendency to remember the final position of a moving object as displaced forward in the direction of motion (11). This measure complements explicit time-to-collision tasks by revealing how the perceptual system automatically anticipates future events (12). Each paradigm taps into partially overlapping but distinct temporal processing mechanisms, together providing a more detailed view on timing abilities in sport.

In the present review, we synthesize research on temporal processing across sports, dividing disciplines into closed-skill, open-skill and extreme sports. The classification of sports skills into “open” and “closed” categories was first introduced by British psychologist Poulton (13) to describe motor tasks in industrial settings, where tasks could be either predictable (closed) or required adaptation to changing conditions (open). This concept was later adapted to sports science by Knapp (14), who applied the open-closed skill continuum to athletic performance, categorizing skills based on the predictability of the environment and the degree of control the performer has over the execution of the skill. We also added the extreme sport category because it reveals the aspect of stress, risk and danger in their influence on time perception - factors we believe warrant separate consideration in athletes. While previous reviews have examined how exercise-related factors such as intensity, duration, and modality influence time perception [for review see (15)], the present review focuses specifically on how time perception differs across sport categories, providing a novel synthesis of temporal processing in closed-skill, open-skill, and extreme sports. Moreover, unlike existing meta-analyses that have concentrated on isolated paradigms [e.g., temporal occlusion (3) or representational momentum (2)], our narrative synthesis integrates findings across explicit and implicit timing tasks, spanning subsecond to suprasecond intervals. By examining behavioral evidence across these sport categories and experimental timing tasks, we aim to provide a comprehensive framework for understanding how athletes perceive, interpret, and interact with temporal information. We also aim to identify current gaps in the study of time perception in sport and to suggest directions for future research. Such insights may inform talent identification, training methodologies, and performance optimization across diverse sport disciplines.

Narrative reviews are well-suited for synthesizing heterogeneous bodies of literature, offering conceptual integration, and identifying patterns across studies that employ diverse methodologies (16). Given the variety of experimental paradigms (duration estimation, production, time-to-collision, motor timing) and sport types (closed-skill, open-skill, extreme) examined in this field, a narrative approach allows us to integrate findings conceptually rather than statistically, highlight recurring themes, and generate new hypotheses for future research.

Relevant articles published from January 2005 to December 2025 were searched. This 20-year timeframe was selected to capture the most recent and methodologically advanced research while ensuring sufficient breadth for a meaningful synthesis. However, we acknowledge that foundational studies were published prior to this period [e.g. (17, 18)]. To address this, we cross-referenced the bibliographies of included articles and, where seminal works were directly relevant to our conceptual framework, we incorporated them into the discussion. A literature search was conducted in two databases, namely PubMed and ScienceDirect using the following keywords: time perception, sense of time, sport, closed-skill sports, open-skill sports, and the names of most popular sports within these categories. Then papers were screened for relevance to the topic. Furthermore, the most pertinent works were identified through an examination of the references cited in the selected publication set. Studies were included if they: were original empirical research published in peer-reviewed journals; involved human participants; used at least one experimental paradigm assessing time perception (duration estimation, production, reproduction, discrimination, time-to-collision, representational momentum, motor timing, temporal resolution, or passage of time judgments); and compared athletes from closed-skill, open-skill, or extreme sports to control groups or examined time perception in relation to athletic expertise. Studies were excluded if they: used only sport-specific stimuli without a general baseline condition unless they compare duration re/production or estimation in sport-specific setting; were case studies; conference abstracts; or non-English publications. We acknowledge that our search strategy did not include PsycINFO, Scopus, or Web of Science, which may have yielded additional relevant articles. However, given the narrative (rather than systematic) aims of this review, and the supplemental cross-referencing of bibliographies from included papers, we believe our search captured the core literature in this domain. Future systematic reviews may benefit from broader database coverage.

The review is structured as follows. Sections 2–4 synthesize findings from closed-skill, open-skill, and extreme sports, respectively. Within the open-skill section, we further subdivide research into racquet and bat sports, combat sports, and team sports, given the distinct temporal demands of each. Section 5 provides a general discussion that integrates findings across paradigms, addresses the question of innate vs. trained abilities, and identifies future research directions. Section 6 concludes with implications for practice and research.

## 2 Closed-skill endurance sports

Rowing, swimming, running and cycling are best described as closed-skill endurance sports. The investigation of time perception during exercise is crucial because athletes frequently rely on internal cues to regulate effort and pace over extended periods. Thus, most studies on time perception in this domain have employed duration production and reproduction tasks over intervals ranging from seconds to minutes.

### 2.1 Effects of exercise intensity and fatigue

Edwards and McCormick empirically demonstrated that exercise intensity significantly distorts the perception of elapsed time, particularly during maximal exertion (19). Their results showed that during short-duration (30s Wingate) and endurance (1200s rowing) self-paced exercise bouts, participants consistently underproduced elapsed time by 10%–30% compared with chronological time with underproduction increased as perceived extension increased. This phenomenon suggests that heightened physical arousal alters neural processing, causing subjective time to accelerate and pass “quicker” than chronological time.

In a later, 2024 study (20), Edwards and colleagues sought to extend these findings to ecologically valid competitive contexts. Participants performed a 30 s time production task before the 4 km cycling trial, during the trial (at either 500, 1500, 2500 m) and 2 min after finishing. To investigate a “competitor effect”, they performed the trial under three different conditions: solo, with a passive opponent avatar, or with an active opponent avatar. They confirmed underproduction of time regardless of whether the activity was performed alone or in a competitive context. However, in this study, time perception changes were independent of the rate of perceived exertion. Notably, the effect of physical exercise was absent in another study, which instead reported sex differences in time perception, suggesting that this factor must be taken into account (21).

In research by Tonelli and colleagues (22) participants engaged in moderate cycling at approximately 40%–60% of maximum heart rate. This moderate physical activity caused an increase in reproduced intervals for all target durations ranging from 0.2 to 3.2 s. However, the effect was significant only for durations shorter than 1 s when percentage differences from the baseline were assessed. Moreover, the direction of the effect depended on interval range: for intervals shorter than 1 s participants overreproduced durations, and physical activity increased this overreproduction, while for intervals longer than 1 s they underreproduced durations, and physical activity decreased this underreproduction. Notably, these alterations in the reproduced interval persisted 15–20 min after exercise completion, even after heart rate had returned to baseline. Weber fraction (perceptual precision) increased during cycling compared to baseline (significantly) and post-cycling (non-significantly) periods. Additionally, the authors included a distance estimation task in their experimental design and found no effect of cycling on this control condition, indicating a specific influence of physical activity on temporal, but not on spatial perception or general cognitive functions.

Petrizzo and colleagues (23) used a duration discrimination task of short durations (0.284–1.268 s) and confirmed the internal time dilation for this time range as a result of physical activity. Notably, Weber fraction and control task performance (numerosity judgement) were also unaffected in this experiment. However, the time dilation dissipated soon after running ceased, in contrast to the persistent effects of previously reviewed results of Tonelli and colleagues (22).

Moore and Olson (24) attempted to isolate the effect of fatigue on time perception from the effects of exercise itself. They recruited healthy, untrained adults (who were not expected to enjoy physical activity as athletes might) to undergo maximal aerobic test. Participants completed a time production task (2–10 s) before and after cycling exercise performed at maximal rate, which induced a high level of perceived exertion. Overproduction of time after the exercise was opposite to the reported speeding of internal time after physical exercise reviewed above (19, 20, 22), and may thus be linked specifically to fatigue. The authors suggest that fatigue shifts attentional focus from timing tasks to the physical sensations associated with fatigue, disrupting normal temporal processing and slowing the internal sense of time. Intense exercise may also induce a negative feeling in non-athletes, which could counteract the effect of physical activity, as emotions are an important factor in subjective time perception (25, 26). Other factors differentiating athletes from non-athletes must also be considered, such as increased competitiveness, motivation for achievements, and adaptation to physical activity tasks.

### 2.2 Sport-specific expertise and temporal calibration

In a study with swimmers and runners Perrone and colleagues (27) employed a duration estimation, time reproduction, finger-tapping tasks, and motor imagery paradigms to assess temporal perception, comparing expert swimmers and runners with non-athletes. They reported greater overestimation of imagined sport actions for controls than for athletes, who were quite accurate. The authors confirmed opposite trends for time dilation and time shrinkage in reproduction tasks in sub- and suprasecond ranges: athletes overreproduced the 500 ms duration and underreproduced the 1,500 ms duration. In the tapping task, swimmers were more consistent throughout the task than other groups, likely due to the sensory-muffled environment of swimming, which may lead these athletes to be more focused on the perception of their internal rhythm.

Research has shown that temporal processing develops in tandem with motor experience, attentional control, and perceptual training. In the work of Tobin and colleagues (28), across three experiments involving elite swimmers, the authors demonstrated that task duration knowledge, formed by hours of training, enhances the accuracy and consistency of time estimations and productions. Swimmers produced more accurate duration when asked to imagine swimming in the pool than when imagining rock climbing for a particular duration (36 s). When swimmers imagine their most trained action (best stroke), their temporal estimations were also more precise than when they imagine their weakest stroke, supporting the idea that increased training improves duration representation of the trained action. Notably, their temporal judgments were not influenced by an interfering cognitive task. Similarly, during real swimming for a particular duration, interfering tasks led to overproduction of duration only when the action was performed in the void context, such as swimming tied to the wall by the stretch cord. The modified context itself also led to overproduction of duration, indicating that duration perception is tightly linked to the context in which the action is usually performed (normal swim). Thus, mental representation of action can be very detailed and specific and contains its duration, which cannot be easily transmitted to actions that are not fully matched to the learnt representation.

Converging evidence from a closed-skill endurance sport comes from subsequent Tobin and Grondin study (29), which examined temporal prediction and estimation in expert and amateur runners. Before a 5-km race, participants predicted their finish time; immediately after the race, they estimated the time they had just run. Experts (provincial/national level) were significantly more accurate than amateurs in both prediction and estimation. Moreover, only the experts showed improved accuracy in the post-race estimation compared to their pre-race prediction, suggesting that extensive motor experience sharpens the ability to not only anticipate performance duration but also update temporal representations online. These results align with the view that highly trained athletes develop a more precise and context-bound representation of task duration, which cannot be fully compensated by general cognitive abilities or lesser experience.

Beyond duration estimation and reproduction, physical exercise also influences duration production—the ability to generate a target interval without external reference. Sysoeva and colleagues (30) examined three tapping tasks in elite athletes, amateur wrestlers and non-athletes: self-paced personal tempo (a measure of preferred internal speed), maximum tapping speed (reflecting motor capacity), and production of a one-second interval (“once per second” tapping). Acute exercise (graded running, swimming practice, or wrestling) increased tapping speed in the maximum and “once per second” conditions, while personal tempo remained unchanged. This effect was independent of sport type, suggesting a robust influence of exercise on two distinct components: general motor speed and the internal representation of conventional time units. Critically, long-term effects were evident in baseline performance: elite athletes (biathletes, cross-country skiers, synchronous swimmers) showed faster maximum and “once per second” tapping compared to non-athletes. The faster maximum tapping likely reflects increased motor efficiency in trained athletes. More revealing are the results for “once per second” production, which taps into central timing mechanisms. The facilitation observed in elite athletes may be explained by an increase in internal pacemaker speed (31) or by heightened awareness of body states (32, 33), both of which can shorten produced intervals (i.e., speed up subjective time). These findings extend the acute effects of physical activity on duration production (19, 20, 22) into the domain of rhythmic motor timing and suggest that repeated exercise-induced speeding may transfer into lasting enhancements in elite performers.

### 2.3 Summary

Collectively, studies on closed-skill sports provide a foundational understanding of how physical activity, fatigue, and sport-specific experience influence time perception—mechanisms that are relevant across all sport types. Three key themes emerge. First, exercise intensity and fatigue systematically alter time perception, with high-intensity exertion typically accelerating subjective time (leading to underproduction) and fatigue potentially slowing it (leading to overproduction). Second, a robust dissociation between subsecond and suprasecond timing is evident, with intervals shorter than 1 s tending to be overestimated/dilated and intervals longer than 1 s underestimated/shrunken—a pattern that persists across experimental paradigms and is modulated by physical activity. Third, sport-specific expertise calibrates internal timing models, as evidenced by the superior accuracy of athletes when estimating or producing durations associated with their trained actions and contexts. These findings underscore the context-dependent nature of temporal processing in endurance sports and highlight the need for ecologically valid research designs that capture the full complexity of pacing and effort regulation in competitive settings.

## 3 Open-skill sports

Open-skill sports unfold in dynamic, often unpredictable environments where athletes must constantly adapt their actions based on external stimuli such as opponents, teammates, or moving objects. These sports place substantial demands on attentional flexibility, anticipatory skills, and rapid temporal decision-making. Whether intercepting a ball in tennis, anticipating a punch in boxing, or navigating complex team strategies in football, athletes are required to perceive and react to time-sensitive information with remarkable speed and accuracy.

### 3.1 Racquet and bat sports

Accurate time perception is crucial in interceptive sports, as athletes must integrate sensory inputs, such as the trajectory of a ball or the actions of opponents, with motor responses to predict when an object will reach a target location. This phenomenon is commonly referred to as anticipation (34). Anticipation of event timing is suggested to rely, at least in part, on an internal sense of time (33).

#### 3.1.1 Time-to-collision and coincidence-anticipation paradigms

Many studies in this domain have employed various versions of the time-to-collision (TTC) or coincidence-anticipation paradigm as will be reviewed below. Zhao and colleagues (35) compared tennis players with non-athletes on their anticipation of a ball bouncing repeatedly on a computer screen—a domain-general time-to-collision task. The bouncing cycle was either 0.667 s (subsecond) or 1.333 s (suprasecond) and the final segment of four bouncing cycles was occluded. Temporal deviations of −25%, 0%, or +25% in speed were introduced in the penultimate segment before occlusion, allowing assessment of both time estimation abilities and participants’ capacity to adapt to changes in ball speed. The key finding of this study was greater variability in estimation for subsecond intervals. This challenges the scalar property, which predicts constant variability if common mechanisms underlay both sub- and suprasecond timing. Further evidence for distinct mechanisms came from opposing directions of bias: participants overestimated the remembered speed for subsecond intervals (pressing the response key later than actual TTC), but underestimated for suprasecond intervals, irrespective of the ball speed. Notably, tennis players exhibited more stable performance than non-athletes across both interval ranges. This phenomenon may be attributed to the dynamic nature of a tennis match, where the speed and trajectory of the ball are constantly changing, necessitating players to continuously adjust to these variations.

Tang and colleagues (36) used similar experimental design but introduced a critical manipulation: a non-beat condition in which only one cycle preceded the occluded final cycle, compared to a beat condition with four regular cycles. In interceptive sports, athletes must remain alert to variations in the repetition rate, making the ability to overcome beat-based expectations crucial. Both tennis players and non-athletes groups performed worse in the beat-based condition, showing greater underestimation (noting that trial duration was >1 s). However, tennis players again demonstrated more stable performance and smaller absolute bias than non athletes. Notably, a significant interaction between conditions and groups emerged for the absolute error: athletes showed a greater advantage over controls in the beat-based than the non-beat condition, indicating superior ability to adjust to changing environmental demands.

However, several studies using the time-to-collision task failed to demonstrate superior performance in athletes, highlighting the context-dependent nature of expertise effects. Chen and colleagues (37) reported no significant association between tennis players’ years of experience and TTC accuracy, suggesting that accumulated practice alone did not systematically reduce timing error or alter the direction of bias. Converging evidence was obtained by Wei and colleagues (38), who found that professional tennis players did not differ from novices in reaction time or TTC error under either 400-ms or 800-ms occlusion conditions. Likewise, a modified TTC task manipulating target and destination speeds revealed no expertise-related differences in timing accuracy among rugby and cricket athletes (39). Across conditions in that study, systematic biases were primarily driven by task kinematics rather than skill level: responses became less delayed (or more anticipatory) with longer occlusion intervals, shifted toward lateness as target speed increased (with constant destination speed), and became more anticipatory as destination speed increased (with constant target speed). Collectively, these findings indicate that TTC performance may be more sensitive to stimulus parameters than to sport-specific expertise *per se*.

#### 3.1.2 Sport-specific calibration of temporal accuracy

The relationship between motor timing and sport-specific experience is further evidenced in coincidence-anticipation timing tasks using the Bassin Anticipation Timer. In these tasks, participants anticipated a light reaching a target and pressed a button to coincide with its arrival. Stimuli were simulated on by a runway of red LEDs that created the apparent continuous motion of an object through sequential switching. Akpinar and colleagues (40) used this device to examine accuracy among athletes from various racket sports at different stimulus velocities (1, 3, and 5 m/s, corresponding to viewing times of 2.2, 0.7, and 0.44 s, respectively). The most accurate performance corresponded to the speeds most characteristic of each sport: tennis player excelled at lowest speed (reflecting the approximately 1 s decision window over 25 m court with ball speed around 27 m/s), badminton players at intermediate speed (reflecting about 0.7 s window over 13 m court with shuttle speeds around 35 m/s), and table tennis players at highest speed (reflecting less than 0.5 s window over a 3 m table with ball speeds around 10 m/s). This fine-tuning to sport-specific demands underscore the role of extensive practice in shaping temporal resolution.

Differences in performance between tennis, table tennis, and badminton players on similar tasks have been replicated across studies (41, 42). Ak & Koçak (41) found that tennis players demonstrated better accuracy in predicting the arrival of a visual stimulus moving at a constant speed of 2 m/s, whereas table tennis players exhibited faster reaction times. Moreover, the playing experience correlated with coincidence-anticipation accuracy in tennis players but with reaction times in table tennis players, pointing to sport-specific adaptation in perceptual-cognitive expertise. Male players also made fewer errors in the coincidence-anticipation timing task than their female counterparts, emphasizing the need to account for sex difference in future research (17, 41).

The ability to control motor timing has also been studied among elderly individuals with varying tennis experience. Lobjois and colleagues (43) compared older tennis players, older non-players, and young adults on a coincidence-anticipation task. While older non-players showed age-related declines in performance, older tennis players were less affected. This result suggests the long lasting and protective effect of training on the effectiveness of motor anticipation.

#### 3.1.3 Implicit timing: representational momentum

Beyond explicit TTC judgments, researchers have examined representational momentum—the tendency to remember the final position of a moving object as displaced forward in the direction of motion—as an implicit measure of temporal prediction. Jin and colleagues (44) compared badminton experts and novices on a representational momentum task using sport-specific stimuli (shuttlecock trajectories). Experts showed larger forward displacement than novices, indicating more robust predictive timing. Critically, a four-years longitudinal training study revealed that novices’ representational momentum increased following badminton training, approaching expert levels. This provides direct evidence that temporal prediction is trainable through sport-specific experience. Similarly, Nakamoto and colleagues (12) found that baseball players exhibited larger representational momentum effects than non-athletes, particularly at shorter presentation durations, consistent with the idea that subsecond timing mechanisms are especially refined in expert athletes.

A recent meta-analysis by Song and colleagues (2) consolidated findings on representational momentum across sports and non-sport domains (e.g., driving, aviation). The analysis revealed a moderate-to-large expertise advantage [Hedges’ g = 0.73, 95% CI (0.54, 0.92)], indicating that experts consistently show greater forward displacement than novices. Notably, this advantage was domain-general: experts outperformed novices even with simple, non-sport-specific stimuli such as dots and geometric shapes. The authors concluded that automatic prediction in visual motion representation is a fundamental perceptual skill refined by expertise, not limited to sport-specific contexts. These findings suggest that representational momentum captures a basic temporal prediction mechanism that is enhanced by extensive perceptual-motor experience, regardless of whether the stimuli are directly related to the expert's domain.

Thus, representational momentum provides a valuable implicit complement to explicit TTC tasks, revealing how the perceptual system automatically anticipates future events. The trainability and domain-generality of this effect underscore its potential as a tool for studying the plasticity of temporal processing in sport.

#### 3.1.4 Motor timing

Motor synchronization abilities have also been investigated in tennis players using a finger-opposition task paced by a metronome at two speeds (0.5 and 2 Hz). Compared to non-players, tennis players exhibited longer touching durations and a higher percentage of correct sequences when the task was performed bimanually, particularly for slower speed (45). These results again demonstrate distinct mechanisms for sub- and suprasecond interval production and emphasize the role of motor expertise in enhancing timing processes within the suprasecond range.

#### 3.1.5 Summary and implications

In summary, time perception plays a critical role for athletes engaged in individual ball sports, contributing significantly to the anticipation of opponents’ actions and the timing of the interceptive movements. The evidence consistently demonstrates that expert athletes exhibit superior temporal accuracy and stability, particularly at speeds and within temporal windows characteristic of their sport. Longitudinal findings suggest that these abilities are refined through training rather than reflecting innate predispositions alone. However, as Avilés and colleagues (46) cautioned, the degree of experimental representativeness critically modulates the observed expert advantage; future research should strive to incorporate ecologically valid designs (e.g., real opponents, full movement responses, competition-relevant speeds) to avoid overestimating or underestimating the role of anticipatory behavior. While our understanding of the precise neurocognitive mechanisms underlying these timing abilities continues to evolve, their significance for performance in interceptive sports is well-established.

### 3.2 Combat sports

Combat sports are characterised by dynamic, technically complex exchanges in which athletes must respond promptly to their opponents’ actions rather than waiting for an attack to be executed. This ability to anticipate attacks is influenced by cues derived from the opponent's movements (47), suggesting that athletes rely on their ability to perceive time at subsecond level, where rapid decision-making is crucial - such as in the momentary window of opportunity to evade an opponent's strike. The recent systematic reviews and meta-analysis by Zhang and colleagues (1, 48, 49) demonstrated that as expertise in combat sports progresses, reaction times and the accuracy of anticipation task performance improve.

#### 3.2.1 Explicit timing: duration reproduction paradigms

Several studies have employed duration reproduction tasks to examine explicit timing abilities in combat sport athletes. Chen and Cesari (50) compared professional fencers (16.2 ± 8.8 years of training experience) with non-athletes on reproduction of visual stimulus in subsecond (300–1,000 ms) and suprasecond (1,100–1,800 ms) ranges. Replicating the general pattern observed regardless of sport experience, all participants reproduced the target interval as longer for subsecond time duration and shorter for suprasecond interval. However, fencers showed smaller reproduction error than non-athletes for subsecond timing (0.220 vs 0.285 ms), while no significant differences emerged for suprasecond range (0.170 vs 0.185 ms). Additionally, timing variability was lower in athletes across both time ranges (0.174 vs 0.211 ms). The authors interpret these findings as evidence for a superior “internal clock” system in elite athletes with particular advantage in the subsecond range - consistent with explosive, interceptive demands of fencing, where actions unfold within a narrow temporal window. Notably, a group of professional pole vaulters (11.2 ± 3.3 years of training) included in the study showed similar performance to fencers, suggesting that long-term athletic training, regardless of the sport type (especially those that require high awareness of body movement at crucial moments), may refine timing mechanisms within short durations.

Jia and colleagues (51), extended this line of inquiry by examining the role of stimulus specificity in duration reproduction. They compared three groups: expert divers (8.06 ± 2.84 years of training experience), amateur wrestlers (2.94 ± 1.63 years of training experience) and non-athletes. Participants reproduced six durations (300, 500, and 700 ms for subsecond time range and 1,300, 1,500 and 1700 ms for suprasecond time range) of two types of visual stimuli: general (geometric shapes) and expertise-related (diving movements). Replicating Chen and Cesari (50), all groups overreproduced subsecond durations and underreproduced suprasecond intervals. However, in contrast to Chen and Cesari (50), no significant differences emerged between amateur wrestlers and non-athletes in either reproduction error or variability for subsecond and suprasecond timing, likely reflecting the wrestlers’ substantially lower training experience (approximately five times less than the fencers in Chen and Cesari's study). This contrast underscores the importance of accumulated training in shaping temporal precision.

Critically, Jia and colleagues (51) demonstrated a sport-specific effect: divers reproduced longer intervals for expertise-related stimuli сompared to general in both subsecond and suprasecond time ranges and this effect increased with training experience among divers (significantly for suprasecond durations). In contrast, amateur wrestlers and non-athletes showed the opposite pattern - shorter reproduction for expertise-related stimuli compared to general stimuli. These results confirm that sport training influences temporal perception by enhancing the efficiency of information extraction from familiar, ecologically valid stimuli.

#### 3.2.2 Speed and distance estimation

The integration of temporal and spatial cues is essential for accurate speed perception in combat sports. Predoiu and colleagues (52) assessed how skill level and sport discipline influence speed and distance estimation in combat sports using the special computerized test. Although no significant differences emerged between disciplines (fencing, karate, boxing, taekwondo), fencers and karatekas showed the highest average scores. Higher skill levels correlated with better estimation abilities.

Similarly, Vasilica and colleagues (53) found that athletes in individual sports, including combat disciplines such as karate and taekwondo, outperformed team sport athletes in speed and distance assessments, highlighting the importance of these perceptual skills in combat sports performance. These findings align with the broader theme that individual, open-skill sports place unique demands on temporal-spatial integration.

#### 3.2.3 Interval timing and pacing

Beyond millisecond and second-range timing, combat sports also require processing of longer intervals—from several seconds to minutes—for effective pacing and energy management. The perception of excessive speed of competition (where the opponent's actions seem “too fast”) often reflects a loss of control and a reduced efficiency, with athletes experiencing a sense of temporal limitation and insufficient time to defend or react (54). Thus, shortage of perceived durations during competition may signal poorer performance.

Interval timing in combat sports involves estimating round durations, typically ranging from three to five minutes. In professional mixed martial arts, fighters must strategically manage energy and tactics throughout a bout, relying on the ability to perceive fight pacing and adjust activation accordingly (55). Effective transitions between low- and high-intensity phases support energy conservation and optimize tactical decision-making. Similar findings have been reported in boxers (56), highlighting the critical role of temporal perception in combat performance.

However, we found only one study that directly assessed the perception of long intervals using chronometric counting in combat athletes (57). In this study Aikido fighters, Quadrato Motor Training (aQMT) practitioners, and non-athletic controls were instructed to press a button when they perceived that specified time intervals (4, 8, 16, and 32 s) had elapsed. No statistically significant differences in the accuracy of time judgment emerged between Aikido athletes and non-athletic controls; both groups showed a tendency to underestimate time. However, aQMT practitioners produced longer time intervals than Aikido athletes, overestimating the target stimulus (statistically significant for the 16-s only). These results may indicate a specific influence of particular training practices on time perception, potentially mediated by enhanced body awareness.

#### 3.2.4 Summary and future direction

Collectively, these studies demonstrate that combat sport athletes develop enhanced perceptual and anticipatory skills with increasing expertise, allowing for more accurate estimation of speed, distance, and timing during dynamic exchanges. Although there are very few studies available, they nonetheless suggest that both rapid temporal judgments and broader interval timing contribute significantly to tactical decision-making and energy management during bouts. Further research is necessary to fully understand the mechanisms underlying time perception across different temporal scales in combat sports and to develop targeted interventions for performance improvement.

### 3.3 Team sports

Football, volleyball, ice hockey and basketball are open-skill team sports that require adaptation to teammates in terms of strategy, tactics and tempo. Athletes in these sports must react to dynamic, unpredictable environments accompanied by constant information flow from teammates and opponents (58). Athletes are required to allocate attention effectively during gameplay under conditions of multiple stimuli (59). These demands may engender distinct patterns of time perception among athletes in open-skill team sports compared to those in individual or closed-skill disciplines.

#### 3.3.1 The phenomenology of “slowness” in elite performance

The ability to perceive and utilize temporal information may represent a fundamental component of the perceptual-cognitive expertise that allows elite athletes to outperform others (60). In a provocative opinion piece, Erren and colleagues suggested that the subjective “slowness” of time may be a key factor separating exceptional athletes such as Lionel Messi and Wayne Gretzky from other players. The authors argue that their dominance in football or ice hockey stems not from superior physical characteristics but from their perception of the game: they experience the game as slower because of their precise and flexible decisions and actions, effectively granting them more time to respond. While this proposal remains speculative due to the absence of direct empirical evidence, it raises important questions about the role of temporal processing in team sport expertise—questions that have begun to be addressed through systematic investigation of specific player populations and task conditions.

#### 3.3.2 Multisensory temporal processing in goalkeepers

Goalkeepers represent a specialized subpopulation of players requiring separate consideration due to their unique task demands. Quinn and colleagues (61) investigated multisensory temporal processing in professional football goalkeepers, outfield players, and non-athletes using a sound-induced flash illusion paradigm. Participants were presented with visual stimuli accompanied by one, two or no auditory beeps. In critical trials, a single flash paired with two beeps typically creates an illusion of two flashes. The temporal binding window (TBW)—the period within which such illusory effects occur—was measured using eight variations of stimulus onset asynchronies ranging from −400 to +400 ms (negative = audio lead; positive = visual lead).

Goalkeepers showed a narrower TBW compared to both outfield players and non-athletes, indicating earlier discrimination of asynchronous stimuli and more precise multisensory temporal processing. The authors suggest that this enhanced ability likely enables goalkeepers to make quicker decisions based on asynchronous multimodal sensory information—a frequent occurrence during matches when visual and auditory cues (e.g., the ball striking a foot and the sound of impact) arrive at slightly different times. This finding demonstrates that specialized positional demands within a sport can shape temporal processing in highly specific ways, even when assessed with domain-general laboratory tasks.

#### 3.3.3 Domain-general temporal abilities in team sport athletes

Beyond position-specific adaptations, researchers have examined whether team sport athletes possess superior domain-general temporal abilities that extend beyond their sport context. Flavell and colleagues (39) investigated prediction motion performance in rugby and cricket athletes using a task that required judging when a moving target would reach a moving destination. This purely perceptual judgment involved no sport-specific cues, allowing assessment of basic temporal prediction mechanisms. Across all experimental conditions, no expertise-related differences emerged between athletes and non-athletes. Converging evidence comes from research using standardized, domain-general anticipation tasks administered within athletic populations. Schumacher and colleagues (62) examined time and movement anticipation in a large sample of youth soccer players using the Time and Movement Anticipation (TMA) test developed by Schuhfried Schuhfried GmbH (Moedling, Austria) (63). In this computerized task, participants observe an occluded moving ball and must estimate both when it will cross a designated line (time deviation) and where it will cross that line (direction deviation). Critically, this study compared performance within the soccer population across age groups (U12 to U23 and professional players) and playing positions (goalkeepers, defenders, midfielders, forwards), rather than comparing athletes to non-athletes. No significant differences in either time deviation or direction deviation emerged across age categories or playing positions. These null findings within a large, talent-selected sample suggest that basic anticipatory timing abilities, when measured with domain-general stimuli, may not differentiate athletes at different stages of development or with different positional demands. For a comprehensive discussion of sport-specific perceptual-cognitive expertise, readers are directed to existing reviews [e.g. (64)].

#### 3.3.4 Summary and implications

To sum up, time perception in team sports remains relatively sparse compared to individual open-skill sports, but several themes emerge. First, the anecdotal claim that elite players experience subjective time slowing—while intuitively appealing—lacks empirical confirmation and requires systematic investigation. Second, goalkeepers in comparison to outfield players and non-athletes, demonstrate enhanced multisensory temporal integration, reflected in a narrower temporal binding window, highlighting how specialized positional demands shape temporal processing.

## 4 Extreme sports

Extreme sports are defined by their high-intensity physical engagement in dynamic natural environments. In contrast to traditional sports, these activities emphasize relational interaction with the environment and are typically not governed by external rules and regulations (65). Engaging in extreme sports is associated with elevated levels of risk and a sense of fear (66). The mechanisms of time distortion in such contexts differ from those in martial arts or team sports. For instance, in mountaineering—where outcomes depend less on pacing or predicting an opponent's actions and more on managing environmental hazards—time perception is heavily influenced by affective state that might vary as a response to increased uncertainty and danger.

The phenomenon of temporal distortion is most evident under conditions of stress that threaten life and have a strong influence on performance in time-restricted contexts (67). From this perspective, in extreme sports, the ability to accurately perceive and process time is critically important. Performing high-speed, highly precise actions under conditions where mistakes can lead to serious injury demands continuous monitoring of highly dynamic environmental changes and rapid adjustment of one's own movements.

### 4.1 Emotion and time perception in extreme sports

Research on time perception in extreme sports has predominantly focused on the relationship between emotional experiences and temporal processing. Campbell and Bryant (68) examined novice skydivers and found that participants who reported higher levels of fear before and during the jump retrospectively estimated the jump duration (objectively about 35 min) as longer. Conversely, those reporting higher excitement before and during the jump perceived its duration as shorter. These results illustrate the interplay between emotions and approach vs. avoidance motivations in shaping subjective time during extreme events.

Stetson and colleagues (69) investigated retrospective duration reproduction in free-fall (objectively about 2 min) and found that participants significantly overreproduced their own fall duration compared to their reproduction of a witnessed fall of another person. The researchers also examined whether this effect might be attributed to higher temporal resolution of visual perception during the frightening event, but found no evidence for its enhancement (participants could not read rapidly alternating digits more accurately during free-fall). They concluded that subjective time dilation in retrospective assessment likely derives from emotional aspects of memory formation than real-time perceptual changes.

Castellà and colleagues (70) examined climbers crossing a high-altitude rope bridge—a high-arousal natural setting. Participants completed a minute production task (press a button when they thought 60 s had elapsed) and provided self-reported arousal, valence, and dominance (feelings of control over a situation) ratings both on the middle of the bridge (high-arousal) and in a safe location afterward (low-arousal). They also retrospectively estimated the time taken to cross the bridge and rated the passage of time. All participants tended to underproduce minutes (indicating subjective time acceleration) at both time points, but this effect was less pronounced after crossing the bridge. Higher arousal correlated with the shorter productions, faster perceived passage of time, and longer retrospective estimates of crossing duration. The decline in arousal, as well as slight increase in dominance and valence from the bridge to the safe location paralleled the reduction in production bias. Valence and dominance were negatively related to passage of time and estimated duration of a trip, though the latter effect did not survive correction for objective duration.

### 4.2 Expertise and temporal control in extreme sports

Utilizing a qualitative, participant-observer approach, Buckley (71) investigated how expert surfers perceive and utilize time across a range of timescales during high-skill physical activity. Drawing on 20 years of participant observation, Buckley demonstrates that surfing expertise involves decision-making processes spanning from long-term planning (days or hours) to subsecond adjustments during wave rides. Crucially, experienced surfers report subjective time dilation during rapid, high-risk maneuvers. This altered time perception enables them to remain consciously aware of attention, body positioning, and decision-making even during split-second movements typically considered automatic. The findings suggest that through extensive practice, surfers enhance their ability to anticipate and process information rapidly, effectively expanding their “mental window” during intense physical action. Buckley argues that such subjective time expansion may reveal fundamental brain mechanisms linking cognition, action, and perception under pressure, making extreme sports a valuable model for studying temporal processing.

Complementing these observations, Weder (72)—drawing on his unique background as an Olympic champion in bobsleigh and a pilot—conducted in-depth qualitative interviews with experts from both extreme speed sports and aviation. His findings on altered perception of time during high-speed action aligns closely with phenomenological reports from surfers. Experts consistently described experience of slow motion, timelessness, and relativity of time and speed, reporting how subjective time perception becomes decoupled from physical time during optimal performance. These accounts resonate with the affective modulation of time judgments documented in extreme sport settings (68, 70) and suggest that altered temporal experience may serve a functional role in enabling experts to perform with precision when the margin for error is minimal.

### 4.3 Summary and implications

Based on existing evidence, we hypothesize that higher expertise in extreme sports—associated with increased control, approach motivation, and lower arousal—may dilate momentary time perception, enabling precise actions. At the same time, retrospective estimates of event duration become more accurate (less overestimation). At the same time, the association of extreme sports with risk may contribute to the experience of more intense emotional states, thereby creating a conducive environment for examining their impact on time perception processes.

## 5 General discussion

**Figure 1:**
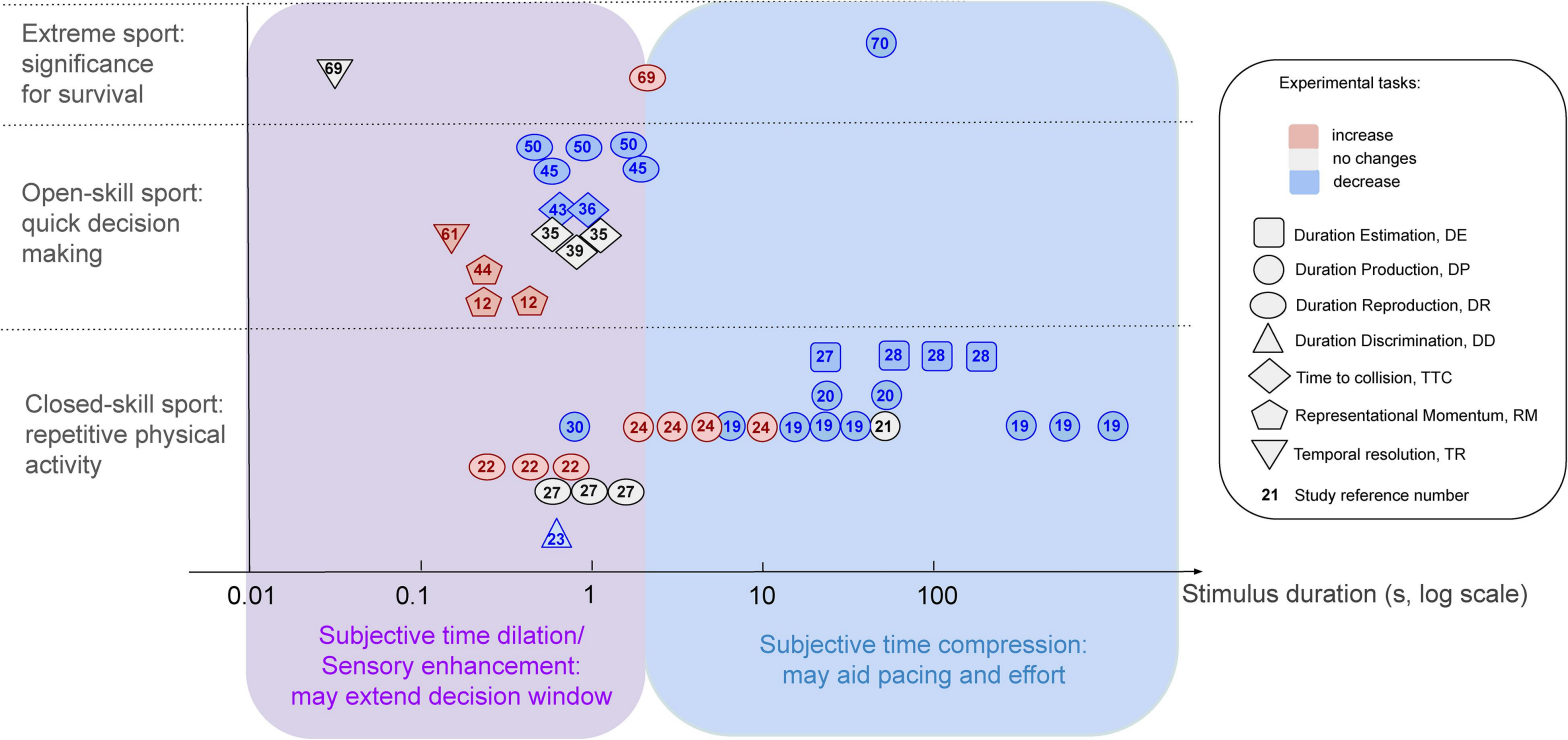
Summary of reviewed studies (*n* = 22) that examined the direction of temporal distortion. Horizontal axis: stimulus duration. Vertical axis: sport category (closed-skill, open-skill, extreme). Color indicates effect relative to control/baseline state or level of expertise: red, increase; blue, decrease; black, no significant change. Markers show experimental paradigms: DE, duration estimation; DP, duration production; DR, duration reproduction; DD, duration discrimination; TTC, time-to-collision; RM, representational momentum; MT, motor timing; TR, temporal resolution. Numbers refer to reference IDs in the main text (see Supplementary Table S1). The red and blue background shading schematically separate two functionally distinct regions: subsecond intervals (<1 s), where duration is typically dilated, and suprasecond intervals (>1 s), where duration is typically compressed. Physical activity and sport expertise enhance both effects.

**Figure 2:**
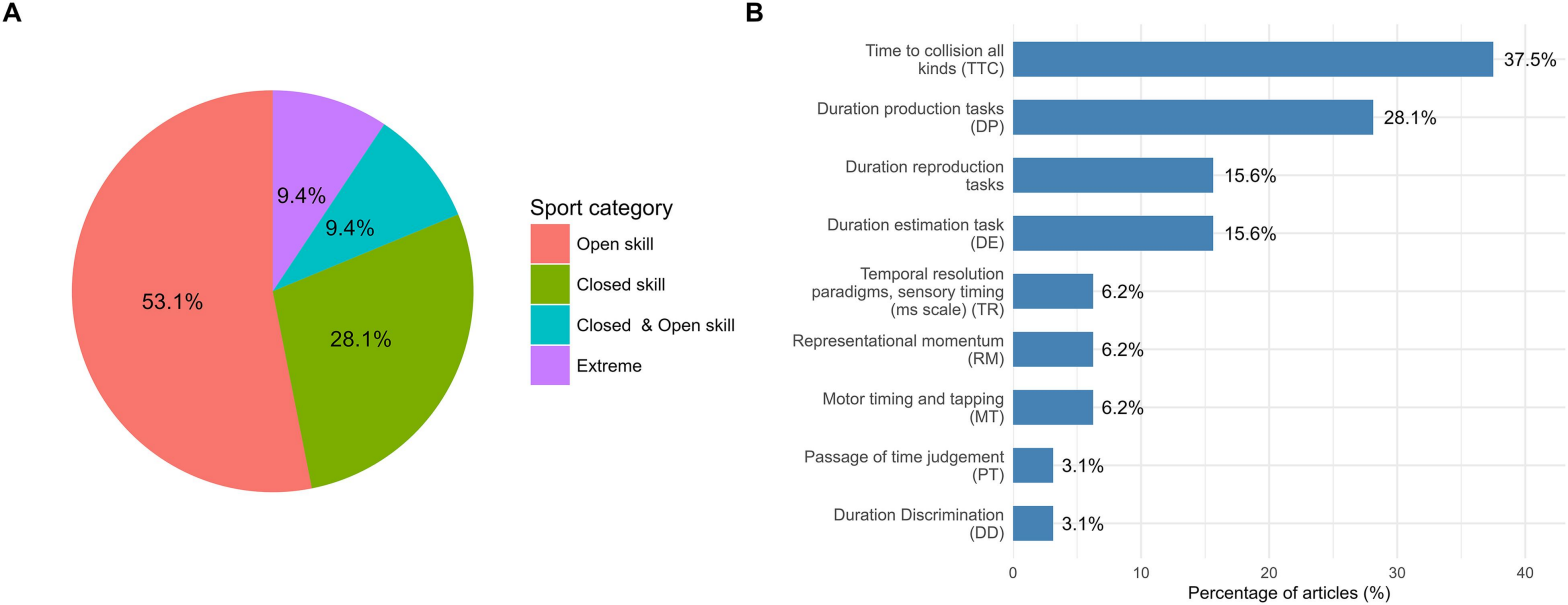
**(A)** distribution of the number of articles by sport category [% of total articles n=32]. Each sector of the pie chart represents a sport category. Percentage values are displayed inside the sectors. **(B)** Distribution of the number of articles by the experimental paradigms [% of total articles n=32]. Horizontal bars show the percentage of publications for each experimental paradigm; values are labelled to the right of the bars.

Figure 1 and Supplementary Table S1 summarize all 32 experimental studies included in this review. In Supplementary Table S1, studies are grouped by experimental paradigm. Within each paradigm, they are organized by sport type (from closed-skill to extreme) and by year of publication (earliest first). Some studies employed multiple paradigms and thus appear in several subsections as separate entries/lines. The paradigms are organized starting with the most subjective measure—passage of time judgement (*n* = 1)—followed by duration estimation (*n* = 5) and production paradigms (*n* = 10), which rely on conventional time units. Next are paradigms dealing with general duration perception, namely duration reproduction (*n* = 5) and duration discrimination (*n* = 1). The final paradigms address more subtle sensory and motor timing: time-to-collision tasks of different types (*n* = 12), which assessed the ability to anticipate the future event through interpolation of speed and trajectory information; motor timing (*n* = 2), measuring synchronization with external stimulation and internal maintenance of those rhythms; representational momentum (*n* = 2), probing implicit temporal prediction through forward displacement in memory for moving objects; and the last subgroup representing the experiments on temporal resolution of perception (*n* = 2), examining the limits of perceptual discrimination. The results are schematically represented on the stimuli duration vs sport type plane on Figure 2. As evident from Supplementary Table S1 and Figures, the distribution of paradigms is not uniformed across sport types: duration estimation and production tasks dominate in closed-skill sports, while time-to-collision are most prevalent in open-skill, particularly interceptive sports. Also open-skill sports lack studies that examine time perception of suprasecond range. This uneven distribution may partly reflect our exclusion criteria, as we did not include sport-specific versions of time-to-collision tasks that incorporate additional ecologically valid variables; such tasks warrant consideration in future reviews. However, an alternative—and more theoretically interesting—interpretation is that this distribution reflects the core thesis of our review: closed-skill sports, which emphasize self-paced interval regulation, are naturally studied with duration production/estimation tasks, whereas open-skill interceptive sports, which demand event-based anticipation, are better captured by time-to-collision paradigms. Thus, the paradigm distribution itself provides convergent evidence for the distinct temporal demands across sport categories. With this methodological context in mind, we now synthesize the main thematic findings emerging from the reviewed literature.

### 5.1 The sub- and supra-second dissociation: a fundamental principle

A consistent finding across all sport types and experimental paradigms is the dissociation between processing of subsecond (<1 s) and suprasecond (>1 s) intervals. Regardless of whether athletes engaged in closed-skill endurance sports (22, 27), open-skill racquet sports (35), or combat sports (50, 51), the same pattern emerged: intervals shorter than one second tended to be overestimated/dilated, while intervals longer than one second were underestimated/shrunken (see Supplementary Table S1, Duration Reproduction and Time-to-Collision sections). This pattern held across explicit timing tasks (estimation, production, reproduction) and implicit paradigms such as time-to-collision, where later responses to shorter occluded intervals may be interpreted as overestimation of remaining time and earlier responses to a longer occluded interval may be interpreted as an underestimation of remaining time (35). The underestimation of time to contact for the future event is evident at longer duration both for athletes or non-athletes, corresponding to general time shrinkage phenomenon (73–75). This trend may be a result of a more severe cost of an overestimation of the time to collision, as it might result in a damage/collision in the real life situation, during running/driving towards the wall or tree. However, in sport, sometimes the accurate estimate of time to contact is crucial that demands the need to overcome the evolutionary more profitable underestimation tendency. Notably, studies of representational momentum—an implicit measure of temporal prediction—reveal that experts show larger forward displacement than novices, particularly for shorter presentation durations (12, 44), consistent with the idea that subsecond timing mechanisms are especially refined in expert athletes.

This dissociation aligns with neurocognitive models positing distinct mechanisms for sub- and supra-second timing. Subsecond timing is thought to rely on automatic, sensory-motor processes mediated by cerebellar-thalamic circuits, whereas suprasecond timing involves higher-order cognitive mechanisms such as attention, working memory, and decision-making, recruiting prefrontal and parietal cortices (6, 8). From an adaptive perspective, the tendency to dilate subsecond intervals may serve to extend the perceptual window during critical moments, reserving additional time for decision-making when rapid responses are required. Conversely, compression of longer intervals may be beneficial for pacing and sustaining effort over extended periods, preventing the subjective experience of time from becoming a source of distraction or fatigue.

This pattern is evident across sport types and paradigms and it does not consistently differentiate athletes from non-athletes in domain-general tasks (39, 62). Therefore, it likely reflects a fundamental property of human time perception, not merely a consequence of athletic training *per se*. However, sport experience does modulate the magnitude and precision of these effects, as discussed below.

### 5.2 Context-dependence and sport-specific calibration

While the sub-/supra-second dissociation appears universal, its expression is modulated by sport-specific demands and expertise level as represented on Figure 2. In closed-skill endurance sports, pacing accuracy improves with training but remains vulnerable to fatigue and exercise intensity. High-intensity exertion typically accelerates subjective time, leading to underproduction of intervals (19, 20), whereas fatigue may slow subjective time (24). As summarized in Supplementary Table S1 (Duration Production tasks), these effects have been documented across cycling, rowing, and running. Tobin and colleagues (28) further illustrated this context-dependence. Swimmers were more accurate when imagining their best stroke than their weakest stroke. They were also more accurate in familiar contexts than in modified ones. Thus, even in self-paced sports, internal timing models are finely calibrated to specific action contexts and are shaped by accumulated motor experience.

In open-skill sports, sport-specific calibration is even more pronounced. Racquet sport athletes excel at speeds characteristic of their discipline: tennis players show superior time-to-collision accuracy at slower speeds, table tennis players at higher speeds [(40) see Supplementary Table S1, Time-to-Collision section]. This fine-tuning extends to the implicit level, as evidenced by enhanced representational momentum for sport-specific stimuli in badminton experts (44) and baseball players (12). Critically, longitudinal evidence demonstrates that such enhancements are trainable: four-years of badminton training increased novices’ representational momentum to approach expert levels (44). In combat sports, fencers with extensive experience show reduced error and variability in duration reproduction, particularly for subsecond intervals (50), while divers exhibit sport-specific temporal biases when processing diving-related stimuli (51). Team sport athletes, particularly goalkeepers, demonstrate enhanced multisensory temporal resolution reflected in a narrower temporal binding window (61), a specialization likely honed by the unique demands of their position.

Collectively, these findings indicate that enhanced time perception in athletes is tightly coupled to sport-specific perceptual experience rather than reflecting a domain-general superiority in temporal processing. When tasks are stripped of sport-specific context, athletes do not consistently outperform non-athletes [(39, 62) see Supplementary Table S1, Domain-General Temporal Abilities]. This pattern underscores the importance of ecological validity in experimental design and cautions against assuming that laboratory measures of basic timing abilities predict real-world athletic performance.

### 5.3 Innate ability or acquired expertise?

A key goal of the application of cognitive science to sport is to improve talent identification and development (64). As such, determining whether enhanced temporal processing can distinguish individuals based on athletic skill level is a critical first step in achieving this goal. However, the evidence reviewed here presents a complex and sometimes contradictory picture.

On one hand, several studies report no differences between athletes and non-athletes on domain-general timing tasks. For example, Flavell and colleagues (39) found no expertise-related differences in prediction motion performance among rugby and cricket athletes, while Ripoll and Latiri (18) observed no expertise effect in a constant-velocity coincidence-anticipation task with table tennis players. Similarly, within athletic populations, performance on such tasks often fails to correlate with expertise level: Schumacher and colleagues (62) found no differences in time and movement anticipation across age groups or playing positions in youth soccer players. Chen and Cesari (50) further demonstrated that tennis players’ years of experience were unrelated to time-to-collision accuracy. These null findings are consistent with the view that fundamental timing mechanisms are relatively stable individual characteristics that do not automatically confer athletic advantage.

On the other hand, when tasks engage the specific perceptual demands of an athlete's discipline, consistent expertise effects emerge. Cross-sectional studies reveal correlations between years of experience and performance across multiple domains: in combat sports, Predoiu and colleagues (52) found that higher skill levels correlated with better speed and distance estimation abilities; in racquet sports, Ak and Koçak (41) reported that playing experience correlated with coincidence-anticipation accuracy in tennis players and with reaction times in table tennis players; and in diving, Jia and colleagues (51) demonstrated that experience modulated the magnitude of temporal biases for diving-related stimuli. Critically, longitudinal evidence directly supports the role of training: Jin and colleagues (44) demonstrated that four years of badminton training enhanced representational momentum in novices, bringing their implicit temporal prediction abilities closer to expert levels. Moreover, the observations and interviewers of athletes involved in extreme and high-speed sports, support the view that it is the expertise that modulates time perception: with greater experience and perceived control, athletes may experience subjective time dilation during performance, enabling more precise actions (71, 72).

This pattern aligns with a recent meta-analysis by Kalén and colleagues (1), who reported that tests of cognitive decision-making skills—a category that primarily included sport-specific anticipation tasks such as time-to-collision judgments with video stimuli—differentiate higher- from lower-skilled athletes more effectively (Hedges' g = 0.77) than tests of basic (g = 0.39) or higher (g = 0.44) cognitive functions. The same meta-analysis also found that sport-specific stimuli yield larger effects than general ones. The authors concluded that domain-general cognitive tests have limited predictive utility for talent identification, whereas tasks capturing the perceptual demands of a specific sport are more sensitive to expertise.

However, an important exception to this pattern comes from studies on representational momentum (RM). A meta-analysis by Song and colleagues (2) found that experts outperform novices on RM tasks even with simple, non-sport-specific stimuli (e.g., dots, shapes), yielding a moderate-to-large effect size (Hedges’ g = 0.73). This suggests that automatic prediction in visual motion representation—a form of implicit temporal processing—may be refined by expertise in a way that generalizes beyond the trained domain. Unlike explicit domain-general cognitive tests (e.g., executive function tasks), RM captures a lower-level perceptual mechanism that appears sensitive to cumulative perceptual-motor experience even with abstract stimuli. Thus, while most domain-general tests have limited discriminatory power, implicit measures of temporal prediction may offer a valuable complement to sport-specific assessments.

Evidence from closed-skill endurance sports further reinforces this pattern. While cross-sectional comparisons show that swimmers and runners produce more accurate temporal reproductions than non-athletes, both athlete groups exhibit comparable timing precision despite different training backgrounds (27), suggesting that general endurance training refines rather than fundamentally alters basic timing mechanisms. Yet, the specificity of motor experience matters: swimmers are more accurate when imagining their best stroke compared to their weakest stroke, demonstrating that even within the same sport, duration representations are calibrated to the most practiced actions (28). Thus, across both open- and closed-skill disciplines, the evidence points to a common principle: basic timing mechanisms may be innate, but their calibration to task-specific demands is highly trainable.

### 5.4 The role of attention, arousal, and affect

Across sport types, time perception is consistently modulated by attention, arousal, and affective state. High concentration narrows attention to task-relevant information and alters the subjective experience of time, producing effects such as flow, timelessness, and slow-motion perception (71, 72). Arousal, whether induced by physical exertion (20, 22) or emotional intensity (68, 70), accelerates subjective time during the event but elongates remembered duration afterward (see Supplementary Table S1, Duration Production and Duration Estimation sections). Valence further modulates these effects: fear leads to longer retrospective estimates, while excitement to the shorter ones (68). For athletes, motivation can buffer the distorting effects of fatigue or discomfort, making training sessions feel shorter and more manageable. Thus, emotional and motivational regulation not only affects athletic performance but also influences adherence to regular exercise. This perspective has practical implications, suggesting that psychological skills training (e.g., arousal regulation, concentration techniques) could enhance athletes’ temporal performance indirectly by optimizing the conditions under which timing occurs.

Another aspect of effective performance in sport is also the ability to enhance/dilate the important moment related to decision-making in high-stakes situations. Extreme sports often involve high levels of physiological arousal and acute stress, which are known to distort temporal processing. Under conditions of imminent danger or rapid decision-making, athletes frequently report the subjective slowing of time, which may reflect heightened attention and accelerated information processing (72). This temporal dilation can provide a functional advantage by allowing the perception of events in greater detail, thereby facilitating timely motor responses in high-risk contexts. However, the reviewed experimental studies only provided direction for examination of such effects, while the effects of enhanced temporal resolution were not confirmed, probably owing to methodological difficulties to study such effects in real-life high-risk situations.

### 5.5 Methodological considerations and future directions

Our review reveals several methodological limitations in the current literature that need to be addressed in future applied research.

First, insufficient integration of different temporal paradigms. Most studies focus on a single task (e.g., only TTC or only duration production), making it difficult to relate findings across temporal scales. Recommendation: Multi-method studies that include both explicit (e.g., duration reproduction) and implicit (e.g., RM, motor synchronization) measures within the same participants would reveal how different timing mechanisms interact.
Second, the underreporting of directionality in time-to-collision (TTC) tasks. Most studies report only absolute error and variability, but the constant error (whether participants over- or underestimate remaining time) is theoretically meaningful and differs between sub- and suprasecond ranges. Recommendation: Future studies should also report constant errors to enable comparison with duration estimation/production paradigms.
Third, the predominance of cross-sectional designs limits causal inference. While cross-sectional comparisons show that experts differ from novices, they cannot determine whether enhanced temporal processing is a cause or a consequence of athletic training. Recommendation: Longitudinal studies measuring temporal processing before and after structured training interventions [e.g., like (44)] are urgently needed to establish causality.
Fourth, low ecological validity in many laboratory tasks and the challenge of transfer. While reviewed domain general laboratory tasks offer experimental control, they may not capture the perceptual-motor coupling of real sports. Furthermore, even when training improves performance on such laboratory tasks, the transfer to actual athletic performance remains uncertain. Recommendation: Applied research should incorporate representative designs (real opponents, full movement responses, competition-relevant speeds) whenever possible, without sacrificing experimental rigor. Virtual reality offers a promising middle ground, but it does not fully solve the transfer problem (76, 77). Systematic comparisons across tasks with varying degrees of specificity and immersiveness could reveal how much representativeness is needed for reliable expertise effects and for successful transfer to competition.
Fifth, individual differences are often neglected. Age, sex, personality, and arousal states systematically modulate time perception, but they are seldom treated as moderators in the reviewed studies. Recommendation: Future research should systematically report and control for these variables, as they may significantly affect both the magnitude and direction of temporal distortions.
Sixth, the neurophysiological underpinnings of sport-specific temporal calibration are poorly understood. Some of the reviewed studies included concurrent EEG registration and reported changes in brain activity that accompanied the performance of time perception tasks (36, 38, 76). While we did not review these results in the current study, this remains a promising direction for future research. Such designs could elucidate the common and distinct mechanisms underlying different aspects of temporal processing in sport. Recommendation: Future research should integrate psychophysiological measures (e.g., heart rate variability, EEG, cortisol) to capture the dynamic interplay of cognitive, emotional, and physiological processes in real time.

Addressing these methodological gaps will make future applied research more robust, comparable, and transferable to real-world sport settings.

### 5.6 A unified framework for temporal processing across sport categories

Our review suggests a unified framework for temporal processing across sport categories, built on the fundamental distinction between subsecond and supersecond duration perception. Applied to sports this distinction translates into two adaptive goals: (i) subsecond intervals during critical moments of decision-making should be dilated to provide more time for optimal action; (ii) suprasecond intervals during prolonged exertion should be compressed to support motivation and reduce perceived effort.

Comparing the three sport categories reveals distinct temporal profiles (Supplementary Table S1 and Figure 2). In closed-skill sports, the primary temporal challenge is self-paced interval regulation, modulated by fatigue, interoception, and exercise intensity. Experts show reduced error in duration production, but high exertion accelerates subjective time (underproduction) while fatigue may slow it. In open-skill (interceptive) sports, the core demand is event-based anticipation, calibrated to sport-specific speeds and cues. Experts exhibit superior time-to-collision accuracy at ecologically valid velocities and enhanced representational momentum for familiar stimuli. In extreme sports, affective states dominate: fear lengthens retrospective estimates, excitement shortens them, and high arousal accelerates prospective time (underproduction). This cross-category synthesis supports an adaptive distortion hypothesis: subsecond dilation may extend the perceptual window for rapid decisions, while suprasecond compression aids pacing and effort management. Extreme sports uniquely highlight how emotion modulates these distortions. Unlike previous reviews [e.g. (15),] that focused on exercise intensity alone, our framework shows that temporal processing in sport is not a unitary skill but a set of context-sensitive mechanisms shaped by environmental predictability, task structure, and affective state.

Implications for talent identification. Basic timing mechanisms may be innate and relatively stable, but they are finely calibrated through extensive practice to meet the specific demands of an athlete's discipline. Domain-general timing tasks have limited predictive value. However, assessments that capture the perceptual and temporal demands of a particular sport—including implicit measures such as representational momentum—could help identify athletes with the potential to excel in fast-paced, interceptive disciplines (64).

Implications for performance and safety regulation. Coaches can use the knowledge that fear lengthens retrospective time estimates while excitement shortens them (68) to help athletes interpret and regulate arousal. In extreme sports, understanding emotion-induced time distortion may improve decision-making under threat.

## 6 Conclusion

Across sport domains, athletes’ temporal processing emerges as a flexible, trainable component of expertise shaped by context, task demands, and psychological state. In closed skill sports, refined internal timing representations support accurate pacing and consistent performance but become vulnerable to distortion under fatigue or high exertion, with experts showing superior temporal consistency through enhanced cognitive control. In open-skill sports, time perception is tightly linked to anticipatory abilities, attentional control, and motor experience, enabling expert performers to excel in estimating time-to-contact and synchronizing with dynamic external cues. Extreme sports further illustrate how high arousal and risk conditions induce time distortions yet experienced athletes develop greater regulation of affective and attentional states to sustain rapid decision-making. Collectively, these findings highlight time perception as a context-dependent and adaptable skill. Understanding its sport-specific mechanisms requires ecologically valid research to inform training that optimizes both performance and safety.
