# Supplementary material for: The mind's clock in sport: a narrative review of temporal processing across athletic disciplines
Source: Front Sports Act Living. 2026 Jul 8;8:1738879. doi: 10.3389/fspor.2026.1738879 (PMC13390061; doi:10.3389/fspor.2026.1738879)
Supplement: Supplementary file 1 [file Table1.docx]

**Table 1. Summary of the main experimental studies.**

| **Source paper** | **Sport category** | **Participants:** n(female=f) aged (range, M (SD) years); experience (M(SD) years), level, regularity of training | **Duration range** | **Details of experimental design** | **Results** |
| --- | --- | --- | --- | --- | --- |
| **Passage of time judgement (PT)** | | | | | |
| Castella et al., 2017 (70) | Extreme (crossing a rope bridge) | Rock climber (n=61(25f) aged 15-59, 31.62(10.93)): Number of times climbed via ferrata, or practiced climbing or caving: 0-9999 times, 14.8% of the whole sample had no prior experience in climbing or similar high-risk sports | N\|A | Passage of time judgements were analysed in relation to affective states (self-assessment manikin test - arousal, valence, and dominance). | ↑Passage of time judgements correlated with ↑arousal and ↓valence and dominance (without correction for objective duration of activity);  ↑Passage of time judgements correlated with ↓dominance (with correction for objective duration). |
| **Duration estimation task (DE)** | | | | | |
| Tobin & Grondin, 2012 (28),  experiment 1 | Closed skill (swimming) | Swimmers (n=28(10f) aged 18-23, NI(NI);  NI; from strong provincial to international level | 66 - 112s | Duration estimation of the imagined 100m swims: 2 with best stroke, 2 with weakest stroke. In each pair, 1 swim was conducted with ongoing cognitive task. | In best compared to weakest stroke:  ↓Estimated/real ratio for 100m swim durations (closer to 1); ↓ABS, narrower range intervals;  no effect of interfering cognitive task. |
| Tobin & Grondin, 2015 (29) | Closed skill (running) | N_wh = 85 (41 f)  Age_wh = 18-66, M=28 (NI)  Expert_runner (n=30); 8.06 (NI); provincial and national level; 4.81 sessions/week, 6.86 hours/week  Amateur_runner (n=55); 2.66 (NI); amateur level; 3.14 sessions/week, 2.82 hours/week | 924 - 1918s | Runners were asked to predict their 5K time before the race and also estimate their running time after completing the race. | Expert vs. amateur runners  ↓ABS both for predicting and estimating their running time. Expert runners had an estimation that was more accurate than their initial prediction. |
| Perrone et al., 2023 (27) | Closed skill (swimming, running) | Runners (n=28(14f) aged 18-25, 20.1(2.16)); 5.86(3.24)) training, expert athletes competing at provincial, and national levels, at least two training sessions per week, 9.84(3.35) hours/week  Swimmers (n=28(14f) aged 18-25, 20.25(2.15)); 10.21(2.48) training; expert athletes competing at provincial, and national levels, at least two training sessions per week, 7.73(5.29) hours/week  Non-athletes (n=28(16f) aged 18-25, 22.53(2.51); University students who do not practice sport regularly and do not compete regularly | about 29s | A temporal estimation of a familiar sport action. Swimmers had to estimate and mentally visualise the time taken to swim freestyle 50m at 80% of their highest speed; runners the time taken to run 200 m at 80% of their highest speed; control group the time for making two flights of stairs. | ↑Overestimation for controls than athletes, who were quite accurate. |
| Campbell & Bryant, 2007 (68) | Extreme (skydiving) | Non-athletes (n=76(37f) aged 15-59, 28(9.6)); who were undertaking their first skydive | several minutes (about 35 minutes) | Estimation of the skydive duration was conducted after landing. Self-reported fear and excitement were assessed before and after the jump. | ↑Estimated duration of a flight correlated with ↑fear and ↓excitement before and during the jump. |
| Castellà et al., 2017 (70) | Extreme (crossing a robe bridge) | Rock climbers (n=61(25f), aged 15-59, 31.62 (10.93)); Number of times climbed via ferrata, or practiced climbing or caving: 0-9999 times, 14.8% of the whole sample had no prior experience in climbing or similar high-risk sports | mean 274+-104s | Estimation of crossing the rope bridge duration were analysed in relation to affective states (self-assessment manikin test - arousal, valence, and dominance). | ↑Estimated duration correlated with ↑arousal and ↓valence and dominance (without correction for objective duration of activity) |
| **Duration production tasks (DP)** | | | | | |
| Tobin & Grondin, 2012 (28),  experiment 2 | Closed skill (swimming) | Swimmers (n=28(10f), aged 18-23, NI); NI, from strong provincial level to international level | 36s | Six 36s-productions were divided in three contexts: swimming normally, swimming with the parachute, and swimming tied to the wall by the stretch cord at either normal or with the secondary task of counting back. | ↑Produced/real ratio in modified context leading to significant overproduction; effect of secondary task only for void context. |
| Tobin & Grondin, 2012 (28),  experiment 3 | Closed skill (swimming) | Swimmers (n=16(8f), aged 18-23, NI); NI, from strong provincial level to international level | 36s | Visualise themselves for  a 36-s target duration either swimming in the university pool or climbing Mount Everest. | Rather accurate production in both tasks (produced/real ratio =1.01), but ↓ABS for swimming condition with the variability in real swimming condition even smaller. |
| Sysoeva et al., 2013 (30) | Closed & Open skill  (biathletes, synchronous swimmers, cross-country skiers, wrestlers) | Biathletes experts (n=15(5f), aged 26(2), Olympic team  Cross-country skiers experts (n=27(9f), aged 25(5)); Olympic team  Synchronous swimmers experts (n=15(15f), aged 21(3); Olympic team  Wrestler amateur (n=9(0f), aged 19(1); University freestyle wrestling group  Non-athletes (n=44(0f), aged 24(6)), who were not involved in regular sport activities | 1s | Three tapping tasks: (1) self‑paced tempo, (2) maximum tapping, (3) production of a “once per second” interval. Tasks performed at baseline and after acute exercise (graded running test, swimming practice, wrestling). Baseline performance compared across groups. | Acute effect of physical exercise:  ↓Produced second  Accuracy ↓in biathletes ↑in amateur wrestlers = in synchronous swimmers  ↓Maximal tapping  =Personal tempo  Between group comparison in baseline:  ↓Produced second (expert athletes vs amateur wrestlers and non-athletes)  ↓Maximal tapping (expert athletes vs non-athletes)  =Personal tempo  ↓Accuracy (synchronous swimmers and skiers experts vs non-athletes and amateur wrestlers) |
| Hanson & Buckworth, 2016 (21) | Closed skill (running) | Runner (n=22(11f) aged 18-23, NI); NI, recreational | 60s | To produce 60s interval by saying "start" and "stop" before, and during self-paced treadmill running. | No significant effect of running;  Men vs female: ↑produced intervals. |
| Edwards & McCormick, 2017 (19) | Closed skill (cycling, rowing) | Non-athletes (n=12(7f) aged 21.1(7.2)); recreationally active | 7.5, 15.0, 22.5, 30.0s (cycling), and 300, 600, 900, 1200s (rowing) | Participants were reminded at regular intervals via an audio recording that they were required to verbally indicate when they perceived 25%, 50%, 75%, and 100% of each bout had been completed. Their responses were audio recorded and time aligned to the task duration for accurate assessment of time production. | ↓Duration production with ↑exercise intensity |
| Perrone et al., 2023 (27) | Closed skill (swimming, running) | Runners (n=28(14f) aged 18-25, 20.1(2.16)); 5.86(3.24)) training, expert athletes competing at provincial, and national levels, at least two training sessions per week, 9.84(3.35) hours/week  Swimmers (n=28(14f) aged 18-25, 20.25(2.15)); 10.21(2.48) training; expert athletes competing at provincial, and national levels, at least two training sessions per week, 7.73(5.29) hours/week  Non-athletes (n=28(16f) aged 18-25, 22.53(2.51); University students who do not practice sport regularly and do not compete regularly | 29 (familiar duration), 17 and 41s | Visualize practising their sport or executing the unpractised sport for three intervals. The results were related to the Vividness of Movement Imagery Questionnaire. | No between group difference with vividness of imagined activity influencing the duration estimation. |
| Moore et al., 2024 (24) | Closed skill (cycling) | Cyclists (n=26(9f) aged 18-40, 25.96 (4.31)); not professional cyclists, nor were they training for a cycling competition in the 6 months prior. | 2, 3, 5, 7, and 10s | Produce duration with a remote keyboard's space bar to start and stop a computer-based stopwatch without feedback at baseline and following the maximal aerobic exercise test on a cycle ergometer and 3 min cool-down. | ↑Overproduction of time (↑difference between the produced and objective duration divided by the produced) after the extensive physical activity. No other effects were significant. |
| Edwards et al., 2024 (20) | Closed skill (cycling) | Non-athletes (n=33(16f) aged 25.9(3.3); recreationally active, no previous experience in performing a cycling time trial | 30 and 60s | Duration production by saying "start" and "stop" before exercise, at 500 m, 1500 m, 2500 m, and post exercise. Three standardized 4-km cycling trials in a randomized order using Velotron 3D software to create a visual, virtual environment representing a solo time trial, a time trial with a passive opponent avatar, and a time trial with an opponent avatar and participant instruction to actively finish the trial before the opponent. | ↓Produced duration during exercise compared to resting and post-exercise measurements.  No difference between exercise conditions or time points while perceived exertion increased throughout the trials. |
| Ben-Soussan et al., 2018 (57) | Open skill (combat) | Aikido group (n=11(7f) aged 48(12)); NI  Quadrato Motor Training group (n=9(5f), aged 48(10)); NI Physically non-active (n=14(7f) aged 48(11)) | 4, 8, 16, and 32s | Duration production (with eyes closed) by pressing a finger button for the required period of time. In addition the complex anti-phase coordination performance was examined. | ↑Produced duration for Quadrato Motor Training practitioners than aikido athletes, which was statistically significant for the 16-s time interval.  No statistically significant differences in the accuracy of time judgment between Aikido athletes and non-athletic controls. |
| Castella et al., 2017 (70) | Extreme (crossing a robe bridge) | Rock climbers(n=61(25f) aged 15-59, 31.62 (3.3)); 65% had no prior experience in crossing the bridge, and 14.8% of the whole sample had no prior experience in climbing or similar high-risk sports. | 60s | Duration production by pressing a stopwatch button and subsequently press it again once they thought that a minute had passed. The task was conducted in the middle of the rope-bridge and 40- minutes after in the safe space together with a self-assessment manikin test. | ↓Production (↑underproduction) in the high-arousal condition paralleled with the ↑in self-reported arousal and ↓valence and dominance by the end of the bridge. |
| **Duration reproduction tasks** | | | | | |
| Tonelli et al., 2022 (22) | Closed skill (cycling) | Non-athletes (n=16(9 f) aged 27.62 (2.55)) | 0.2, 0.4, 0.8, 1.6, 3.2s | The reproduction of the rectangle duration by the space bar press. The task was performed 3 times: at baseline, while sitting comfortably on the chair, during physical activity while cycling with 40–60% of the maximum heart rate and around 15min after cycling, when the heart rate had returned to the basal rate.  Distance estimation task as a control condition. | Durations <1s were overreproduced, while >1s were underreproduced.  ↑Reproduced duration during and post cycling relative to baseline for all durations. The effect was significant only for <1s durations when percentage of difference from the baseline were assessed.  ↑Perceptual precision during cycling compared to baseline and post-cycling periods.  No effect for distance estimation task. |
| Perrone et al., 2023 (27) | Closed skill (swimming, running) | Runners (n=28(14f) aged 18-25, 20.1(2.16)); 5.86(3.24)) training, expert athletes competing at provincial, and national levels, at least two training sessions per week, 9.84(3.35) hours/week  Swimmers (n=28(14f) aged 18-25, 20.25(2.15)); 10.21(2.48) training; expert athletes competing at provincial, and national levels, at least two training sessions per week, 7.73(5.29) hours/week  Non-athletes (n=28(16f) aged 18-25, 22.53(2.51); University students who do not practice sport regularly and do not compete regularly | 0.5, 1.0, 1.5s | Reproduction of the target stimulus duration by pressing the space bar when a similar tone is stopped. Each duration was repeated 12 times in random order, following three practice trials. | ↓Underreproduction for runners compared to swimmers in the 1.5s interval; all groups overreproduced 0.5s interval and were accurate for 1s interval. |
| Chen & Cesari, 2015 (50) | Open skill (fencing, pole vaulting) | Fencers (n=15(10f) aged 27.5(8.0)); 16.2(8.8)); national level or higher; NI  Pole-vaulters (n=12(3f) aged 26.8(4.0)); 11.2(3.3) years; national level or higher; NI  Non-athletes (n=23(13f) aged 28.0(4.7); no competitive sports experience | 0.3-1.8s | Reproduction of visual stimulus that flashed for a specific temporal duration by pressing and releasing the space bar of a computer keyboard. | ↑Reproduced duration for <1s duration; ↓Reproduced duration for >1s duration for all groups;  ↓Percentage of errors for athletes than nonathletes, but significant only in <1s duration;  ↓Variability for athletes compared to non-athletes for both time ranges. |
| Jia et al., 2020 (51) | Open skill, Closed skill (wrestling, diving) | Divers (n=27(15f) aged 10–17, 14.00 (3.09)); 8.06(2.84); National Level 2 or higher; NI  Wrestlers (n=27(3f), aged 15–18, 16.52(1.22)); 2.94(1.63); provincial-level, NI  Non-athletes (n=27(13f), aged 12–13, 12.56(0.51)); students with no competitive sports experience | 0.3- 1.7s | Reproduction of visual stimulus by pressing and releasing the space bar of a computer keyboard. Two types of stimuli were employed in this task: general stimuli (such as square, a circle, a star, and a triangle) and the expertise-related stimuli (four diving movements). | ↑Reproduced duration for <1s duration; ↓Reproduced duration for >1s duration for all groups;  Variability of timing was similar across time range and groups; Reproduction error was similar between amateur wrestlers and nonathletes for all durations.  ↑Reproduced duration for expertise-related stimuli сompared to general in both subsecond and suprasecond time ranges in divers, while the other samples showed the opposite pattern. This effect positively correlated with ↑training experience among divers (only for >1s duration).  ↑Precision of duration perception for <1s duration for general stimuli for divers compared with the wrestlers and nonathletes. |
| Stetson et al., 2007 (69) | Extreme (free falling) | Non-athletes (n=20(NI), NI) | 2.49s | Reproduction of the free fall duration was compared to reproduction of a witnessed fall of another person. | 36% ↑ reproduced duration of own fall than of a witnessed fall, however still slight undereproduction. |
| **Duration Discrimination (DD)** | | | | | |
| Petrizzo et al.,2022 (23) | Closed skill (running) | Non-athletes (n=15(8 f) aged 27.3(6.4)) | 0.284- 1.268s | Reference stimuli of 0.6s were presented 6 times at the beginning and then testing stimuli were presented. Participants have to say if the presented duration is the same or different from the reference. All stimuli were visual squares. There was a training session with feedback that ended when 80% of correct responses were made. Then the test is performed while standing or running on a treadmill or after running. A numerosity judgment task was performed as control. | Point of subjective equality shifts to shorter values during running in comparison to "before" and "after".  Weber fraction is not changed in both conditions.  No changes in numerosity judgement in both sessions. |
| **Time to collision all kinds (TTC)** | | | | | |
| Lobjois et al., 2006 (43) | Open skill (tennis) | Tennis players (n=3*10(0f) aged 20-30 23.5(2.7)), 60-69(65.7(3.4)), 70-79(76.4(3.4)), NI(NI); >10 years of experience (14.7-34.5, 26.3(NI)); recreational, non-expert, 2–4 hr/week  Non-athletes (n=3*10(0f) aged 20-30 (25.7(3.8)), 60-69(65.3(2.1)), 70-79 (73.7(2.5)), NI(NI); students and retired individuals with more than half reported engaging in physical exercise (e.g., walking, swimming) for 1–4 hr/week. | Trials varied in stimulus velocity: slow (1.77m/s), intermediate (3.55m/s), and fast (5.33m/s). The viewing time was identical in all conditions (750ms). | The participants' task was to press a button to coincide with the arrival of a moving stimulus on the Bassin Anticipation Timer. To start the trial, participants pressed a button. | CE:  young tennis players=young non-players=  old tennis players<old non-players (especially at higher speed)  VE:  tennis players<non-players irrespective of age |
| Ak & Koçak, 2010 (41) | Open skill (tennis, table tennis) | Tennis players (n=107(55f) aged 10-14, 12.2(1.0); 3. (1.5); NI; NI  Table tennis players (n =101(42f) aged 10-14, 12.4(1.2); 3.7(1.2); NI; NI | 2 m/s | The participants' task was to anticipate a light reaching a target and press a button to coincide with its arrival on the Bassin Anticipation Timer. The stimulus was preceded by a vocal warning, followed by the stimulus. | Tennis vs table tennis players:  ↓Error in the coincidence-anticipation timing task  ↑Reaction time  The playing experience correlated with coincidence-anticipation accuracy in tennis players and with reaction times in table tennis players  Male vs female players:  ↓Error in the coincidence-anticipation timing task. |
| Akpinar et al., 2012 (40) | Open skill (tennis, badminton, table tennis) | Tennis players (n=30(15f) aged 10-15, 12.4(1.4)); 3.65(0.46); NI; >3 days/week  Badminton players (n=30(15f) aged 10-15, 12.5(1.4)); 3.5(0.5); NI; > 3 days/week  Table tennis players (n=30(15 f) aged 10-15, 12.4(1.16)); 3.5 (0.49); NI; >3 days/week | Stimuli were presented at three velocities: low (1 m/s), moderate (3 m/s), and high (5 m/s) with 2.2 s, 0.7 s, and 0.44 s of viewing time, respectively. | The participants' task was to anticipate a light reaching a target and press a button to coincide with its arrival on the Bassin Anticipation Timer. | ↓ABS and VE under the trained speed: tennis (1 m/s), badminton (3 m/s), table tennis (5 m/s) |
| Vasilica et al., 2013 (53) | Open and closed skill | Individual sports (Tennis, Gymnastics, Karate, Taekwondo); (n=32(16f) aged 20-24, NI (NI)  Team sports (Football, Handball, Basketball) (n=32(16f) aged 20-24, NI (NI)  Non-athletes (n=32(16f) aged 20-24, NI (NI); do not conduct systematic physical activities) | NI | The participants' task was to press a button when three moving targets aligned simultaneously in a common space. The stimuli consisted of three red target circles moving clockwise on concentric orbits, accompanied by three distractors moving counterclockwise at varying speeds. | Task performance assessed by multiple parameters:  Athletes (from team and individual) are better than non-athletes.  Individual sport athletes are better than team sport athletes. |
| Flavell et al., 2018 (39) | Open skill (football, cricket) | Rugby (n=19(0f), club players;  Cricket (n=24(0f), club players;  Cricket (n=16(16f), Members of the England's national women's cricket team;  Non-athletes (n=49(20f): Students who were not routinely engaged in ball sports. | Target occlusion period was 0.75, 1, or 1.25 s. Target and destination speed was 10, 15 or 20 °/s. | The participants' task was to estimate when an occluded moving ball would reach its destination. The destination either moved away from (Chase) or toward (Attack) the moving target. | СЕ was unaffected by athletes’ group (as determined by sport, sex, and expertise level) ↓CE (less late/increasingly early) with longer occlusion intervals, higher destination speed in all participants for conditions.  ↑CE (less early/increasingly late) with target speed increased in Chase condition. |
| Schumacher et al., 2018 (62) | Open skill ( (Soccer) | Soccer player (n=178(0f) aged 10-33, 16.2 (4.8)); highly talented players from a German 2nd league club's youth academy (U12-U23) and professional team, playing at the highest and 2nd highest national competition levels for their age | NI | The participants' task was to estimate when an occluded moving ball would reach its destination by Time and Movement Anticipation (TMA) by Schufried GmbH | Time deviation and direction deviation in in the anticipation test show no group differences for age or position |
| Dubey et al., 2022 (42) | Open skill (racquet sports) | Tennis players (n=10(0 f) aged 18-26, NI (NI); "good and sound level of skill"; "regular players"  Badminton players (n=10(0f) aged 18-26, NI (NI); "good and sound level of skill"; "regular players"  Table Tennis players (n=10(0f) aged 18-26, NI (NI); "good and sound level of skill"; "regular players" | 2 m/s | The participants' task was to anticipate a light reaching a target and press a button to coincide with its arrival on the Bassin Anticipation Timer. The warning signal preceded the stimulus with a random interval in the range of 0.5-3 seconds. | Test scores:  tennis players < table tennis players < badminton players |
| Chen et al., 2022 (37) | Open skill (badminton) | Badminton players (n=28(14f) aged 21.35(2.65)), 12.2(3.75); played in professional  badminton clubs, most of them listed  in the Badminton World Federation rankings | NI | The participants' task was to estimate when an occluded moving ball would reach its endpoint. Additional task on compensatory tracking task performance (control of drifting ball). | No significant correlation between years of training and TTC accuracy, while compensatory tracking task performance correlated with years of training. |
| Predoiu et al., 2024 (52) | Open skill (boxing, fencing, karate, taekwondo) | Combat sport athletes (n=66(21f) aged 18-26, 21.22(2.90)); >2 years of competitive experience and having > 18 years old represented inclusion criteria for this study | NI | The participant's task was to track red balls on the screen, which moved at different speeds and trajectories, and to press a button when the balls lined up. | Performance did not differ statistically significantly between sports. More sport expertise was associated with better performance. |
| Tang et al., 2025 (36) | Open skill (tennis) | Tennis players (n=28(10f) aged 18-26, 22.21(3.12)); 6.50(3.71); Competitive (awards at regional or higher levels); 8.76(3.99) hr/week  Non-athletes (n=28(10f) aged 19-27, 20.59(3.17)); did not engage in regular exercise | The ball moved in a standard 1 s cycle, with temporal deviations of -25%, 0%, or +25% in the penultimate segment's speed. | The participants' task was to estimate when an occluded moving ball would reach its endpoint under two contexts (beat and non-beat). In the non-beat context movement phase consisted of 2 cycles. In the beat condition, the movement phase consisted of 4 cycles. In both contexts the final segment was occluded and the penultimate segment's speed provided the basis for predicting the ball's position. | Tennis players vs non-athletes:  ↓ABS in both non-beat and beat context, while greater effect in beat context (tennis player did not have attenuation of ABS in beat condition that was present in non-athletes)  ↓Delayed response ratio, the ratio of the number of trials that overestimate time to the number of total trials) (fewer delayed responses) |
| Zhao et al., 2025 (35) | Open skill (tennis) | Tennis players (n=28(11f) aged 23.11(2.38)); 7.34(4.90); actively competing at various levels (regional or higher); ~8.76 (3.99) hr/week  Non-athletes (n=27(7f) aged 22.19(2.54)); no tennis experience; lacked any background in competitive sports; did not partake in routine physical activity | The standard cycle was either 0.667s or 1.333s with temporal deviations of -25%, 0%, or +25% in the penultimate segment's speed. | Similar as (36) beat condition. | ↑CE for < 1 s durations  Overestimation of the remembered speed (pressing key later) for <1s duration; underestimation for >1s duration, irrespective of the ball speed  Tennis players vs non-athletes:  ↓VE  =CE, ABS |
| Wei at al., 2025 (38) | Open skill (tennis) | Tennis players (n=17(10f) aged 10.76(2.56)); Qualified as a National Player in second grade or above; Practiced more than three times a week for two or more hours in the last 2 years.  Novice (n=17(9f) aged 21.2(2.14)); College students who took an 18-week tennis course (1.5 h/week) but did not meet any criteria for being a tennis expert. | The duration of movement before occlusion was 1s, the duration of occlusion was 0.4 or 0.8 s | The participants' task was to estimate when an occluded approaching sphere would reach its destination (motion-in-depth perception). Concurrent EEG. | No significant main effect of either group (expert vs novice) or occlusion duration on TTC accuracy was found. However, the expert  group showed shorter neurophysiological response (P1 latency under the TTC1 (actual time-to-collision is 0.4  ms) condition). |
| Representational momentum (RM) | | | | | |
| Nakamoto et al., 2015 (12) | Open skill (baseball) | Baseball players (n=9(0f) aged 20-22); the official college baseball team (> 10 years playing experience). Participants generally spent 20 h/week practicing or playing actual games.  Non-athletes (n=9(0f) aged 20-22); college students who played baseball but had not received any special baseball training. | The slow (10 m/s) and fast (15 m/s) conditions with total time of 0.4 and 0.266 s, respectively | Trackway of 200 LEDs that simulated the linear motion of objects.  RM task: judge the moment of vanishing by naming the respective card.  TTC task: Press a button coinciding with the arrival of an apparent moving target at the end of the trackway.  Occluded and non-occluded trails presented randomly (40 trails each) | ↑RM (further displacement in the direction of motion) for baseball players compared to non-athletes.  ↓CE in TTC in baseball players compared to non-athletes in fast condition.  RM and CE were strongly correlated in the fast velocity condition in both groups, whereas in the slow velocity condition, a significant correlation appeared only in baseball players. |
| Jin et al., 2017 (44) | Open skill (badminton) | Experiment 1 Cross-sectional:  Badminton experts (n=20(10f) aged 22.48(4.57)); >5; qualified as a national player (second grade or above); >3 2-hour training sessions/week for the last 2 years.  Novices (n=19(9f) aged 20.90(4.19)); students with no professional training in any ball sports Experiment 2 (Longitudinal):  Novice trainees (n=12(6f) aged 14.42(0.79); < 10 months of amateur training; With 4 years of professional badminton training follow-up; Practiced at least three times per week and 2 hours/practice for the 4 years. | The presentation of each of the four rectangles was 0.25 ms with rotation 20° | The main task required participants to judge whether a briefly presented probe rectangle matched the orientation of the third rectangle in a sequence that created an illusion of continuous rotation. | Experiment 1  ↑RM for badminton players than for novices.  Experiment 2  ↑RM after four years of systematic badminton training. |
| **Motor timing and tapping (MT)** | | | | | |
| Perrone et al., 2023 (27) | Closed skill (swimming, running) | N_wh = 84 (44 f)  Age_wh = 18–25, M = 20.98 (2.51)  Runners (n=28(14f) aged 18-25, 20.98(2.51); 5.86(3.24), expert athletes competing at provincial, and national levels, at least two training sessions/week, 9.84(3.35) hours/week  Swimmers (n=28(14f) aged 18-25, 20.98(2.51); 10.21(2.48), expert athletes competing at provincial, and national levels, at least two training sessions/week, 7.73(5.29) hours/week  Non-athletes (n=28(16f) aged 18-25, 20.98 (2.51); University students who do not practice sport regularly and do not compete regularly | 0.6 and 0.8s | In each trial, participants first synchronized 18 keypresses with a regular stimulus. After the stimulus stopped, they continued producing 36 keypresses at the same pace, aiming for accuracy. A single practice trial was given for familiarization. Participants were instructed not to use mental strategies, and no feedback was provided. | While all participants improved over time, swimmers were more consistent throughout the task. Runners and non-athletes showed greater performance gains in the second half of the test. |
| Bisio et al., 2021 (45) | Open skill (tennis) | Tennis players (n=20(7f) aged 10-18, 13.75(2.47); 4.80(2.02); local tennis teams; 2.35(0.61) hours/week  Non-tennis players (n=22(9f) aged 11-18, 14.32(2.42) | 0.5, 2s | Finger-opposition movements in sync with a metronome during two tests: a bimanual coordination test comparing motor performance between the dominant and non-dominant hands, and a movement lateralization test comparing the dominant hand’s performance in single-hand vs. bimanual tasks. Motor performance was assessed by movement strategy and movement accuracy, both temporal and spatial. | In general in 0.5 than 2 Hz task:  ↑touch duration;  ↑correct response by right hand in a bimanual task.  Tennis players vs controls:  ↑touch duration, especially in 0.5 Hz task;  ↓inter-tapping interval (duration reproduction);  ↑correct response by right hand in a bimanual task. |
| **Temporal resolution paradigms, sensory timing (ms scale) (TR)** | | | | | |
| Quinn et al., 2023 (61) | Open skill (football) | Football (goalkeepers) (n=20; aged 20-37, 26.8(5.6))  Football (field players) (n=20 aged 19-36, 25.1(4.9)), professional, NI  Non-athletes (n=20; aged 20-37, 24.5(4.8)) | 0.116- 0.192s | Participants were required to report how many flashes they perceived and were instructed to ignore the auditory beeps, which were irrelevant to the task. On each trial, the visual flash stimulus/stimuli could be accompanied by one, two or no auditory beeps. In conditions containing two flashes or two beeps, auditory and visual stimuli were separated by one of eight stimulus onset asynchronies (-400 ms, -200 ms, -150 ms, -100 ms, 100 ms, 150 ms, 200 ms, 400 ms). | Goalkeepers had a narrower temporal binding window and were less susceptible to the illusion as compared to the outfield and control groups. |
| Stetson et al., 2007 (69) | Extreme (free falling) | Non-athletes (n=20 (NI), NI | 0.03s | The determination of the correct value in a task with rapidly alternating stimuli (the digit alternated with its negative image) on the ground and during the flight. | No hypothesized increase in temporal resolution, as about 20% of correct responses at increased (36%) rate were reported. |

Table notes:

CE - constant error (the difference between the real and estimated time-to-contact);

VE - variable error (the standard deviation of the errors and indicated the response dispersion);

ABS - absolute error (the absolute error between the estimated and actual time-to-contact)
